# Reductions in sugar sales from soft drinks in the UK from 2015 to 2018

**DOI:** 10.1186/s12916-019-1477-4

**Published:** 2020-01-13

**Authors:** L. K. Bandy, P. Scarborough, R. A. Harrington, M. Rayner, S. A. Jebb

**Affiliations:** 1 Centre on Population Approaches for NCD Prevention, Nuffield Department of Population Health, University of Oxford, BDI Building, Old Road Campus, Oxford, OX3 7FZ UK; 2 Nuffield Department of Primary Health Care Sciences, Radcliffe Primary Care Building, Radcliffe Observatory Quarter, University of Oxford, Woodstock Road, Oxford, OX2 6GG UK

**Keywords:** Public health policy, Sugar reduction, Reformulation, Industry, Soft drinks

## Abstract

**Background:**

The consumption of free sugars in the UK is more than double the guideline intake for adults and close to triple for children, with soft drinks representing a significant proportion. The aim of this study was to assess how individual soft drink companies and consumers have responded to calls to reduce sugar consumption, including the soft drink industry levy (SDIL), between 2015 and 2018.

**Methods:**

This was an annual cross-sectional study using nutrient composition data of 7377 products collected online, paired with volume sales data for 195 brands offered by 57 companies. The main outcome measures were sales volume, sugar content and volume of sugars sold by company and category, expressed in total and per capita per day terms.

**Results:**

Between 2015 and 2018, the volume of sugars sold per capita per day from soft drinks declined by 30%, equivalent to a reduction of 4.6 g per capita per day. The sales-weighted mean sugar content of soft drinks fell from 4.4 g/100 ml in 2015 to 2.9 g/100 ml in 2018. The total volume sales of soft drinks that are subject to the SDIL (i.e. contain more than 5 g/100 ml of sugar) fell by 50%, while volume sales of low- and zero-sugar (< 5 g/100 ml) drinks rose by 40%.

**Conclusion:**

Action by the soft drinks industry to reduce sugar in products and change their product portfolios, coupled with changes in consumer purchasing, has led to a significant reduction in the total volume and per capita sales of sugars sold in soft drinks in the UK. The rate of change accelerated between 2017 and 2018, which also implies that the implementation of the SDIL acted as an extra incentive for companies to reformulate above and beyond what was already being done as part of voluntary commitments to reformulation, or changes in sales driven by consumer preferences.

## Introduction

National surveys show that the consumption of free sugars in the UK is more than double the guideline intake for adults and close to triple for children aged 4–10 and 11–18 years [1]. Soft drinks have been a major source of free sugars for many years [2] and currently account for 21% (57 g/day) and 33% (67 g/day) of the total free sugar intake in adults and children, respectively [1]. Following a report from the Scientific Advisory Committee on Nutrition which recommended a lower target of 5% dietary energy from free sugars [3], the UK Government challenged the food industry to reduce the sugar content of foods by 20% by 2020 [4]. Furthermore, in March 2016, it announced a three-tiered levy on sugar-sweetened soft drinks, which was implemented in April 2018 and is the first soft drink tax in the world to have multiple tiers designed to drive reformulation [5]. Products that contain more than 8 g sugar per 100 ml are now taxed at 24 pence per litre and products that contain 5–8 g/100 ml are taxed at 18 pence per litre. Products with less than 5 g sugar per 100 ml are not subject to the tax [5]. Pure, unsweetened fruit juice and flavoured milk drinks (amongst other smaller categories) are excluded from the levy. The challenge to the food industry to reformulate their products and the introduction of the tax have been accompanied by a large public awareness campaign, particularly as part of an initiative called Change4Life [6] as well as increased attention to sugar-related harm in the mass media [7].

We aimed to assess the sugar content of soft drinks, both at the aggregate category and company level. Using a novel method that links nutrient composition data and volume sales of soft drinks, we have analysed how shifts in consumer demand and changes made by individual soft drink companies have impacted on the total volume and per capita volume sugar sales from soft drinks. We focus on the period from 2015 to 2018 which spans a period of growing action on soft drinks, including changes in consumer awareness and behaviour, voluntary reformulation characterised by public pledges from some companies, the announcement of a soft drinks industry levy (SDIL) in March 2016 and its implementation in April 2018.

## Methods

We estimated the total contribution of sugar to the food supply for all of the companies in the soft drinks sector by pairing brand-level sales data with individual product-level nutrient composition data on an annual basis for four consecutive years from 2015 to 2018.

### Data types and sources

We identified soft drink manufacturers in the market in 2018, including retailers who manufacture their own-label products, using data obtained from Euromonitor. Euromonitor is a private research company that provides sales data collected from primary and secondary data sources, including store audits, interviews with companies, publicly available statistics and company reports [8]. Its coverage includes supermarkets, convenience stores, markets, vending machines, restaurants and fast food outlets. We calculated the total annual sales for all brands across eight types of soft drink, as defined by Euromonitor: bottled water, carbonates, concentrates, 100% juice, juice drinks, energy drinks, sports drinks and others. Bottled water and 100% juice were included, despite being excluded from the tax, to provide a picture of how the market share of these products may have changed compared to sugar-sweetened drinks. Milk and milk-based drinks, hot drinks and drinks containing alcohol were excluded.

A brand was defined as a set of products that have the same name and are manufactured by one company. One brand of soft drink may have multiple variants. For example, the company Britvic manufactures a number of different brands, including Pepsi, Pepsi Max, Lipton Ice Tea and Robinsons. Within a single brand, there may be a number of different products, for example, Lipton Ice Tea with Peach and Lipton Ice Tea with Lemon.

The product-level nutrient composition data of individual soft drinks were provided by Brand View, a private analytics company that collects product information, including nutrient composition data, by scraping from websites of the three leading UK retailers: Asda, Sainsbury’s and Tesco. These data were scraped on the same date (13 December) for four consecutive years (2015, 2016, 2017 and 2018).

### Matching the sales data and composition data

To estimate the sales-weighted mean sugar content of soft drinks in the UK market and to calculate the total amount of sugars sold by each company, we matched the Euromonitor brand-level data with the Brand View product-level data. When one brand had multiple variants, the mean sugar content for the variants was calculated and paired with the sales data. For example, the 2018 volume sales data for the brand Lipton were matched with the mean nutrient information collected from five individual products with different flavours and/or sizes.

Corresponding nutrient composition data could not be found for 23 brands in the sales database. For 12 of these brands, it was because they were not sold in the supermarkets included in the Brand View database. For these brands, the nutrient composition data were sourced online from the brand website in early 2019, and this data was used for all four years. The remaining 11 brands (constituting < 1% of total volume sales) did not have matching composition data in the given time period and were removed from the dataset. Euromonitor classifies a number of small and local brands under the umbrella of “others”, and these products, representing 21% of the total volume sales of soft drinks, were excluded from all analysis.

### Statistical analysis

The total volume of sugars sold (tonnes per year) and the sales-weighted mean sugar content were calculated for each company. Per capita results were calculated using annual population estimates from the Office for National Statistics (ONS) [9], and per day results were calculated by dividing by 365.

Each of the products was categorised according to their sugar content in relation to the tax payable under the SDIL. Products that are subject to the SDIL and contain > 8 g/100 ml sugar were categorised as “high-sugar” and those between 5 and 8 g/100 ml as “mid-sugar”. Products with less than 5 g of sugar and not subject to the SDIL were split into two discrete groups: “low-sugar” if they contained 0.1–5 g/100 ml sugar and “zero-sugar” if they contained < 0.1 g/100 ml sugar. Plain bottled water was assigned its own category, “bottled water”. Fruit juices with no added sugar are excluded from the tax and were categorised as “exempt”.

Neither the sales nor composition data were normally distributed, so we calculated the mean and median sugar content and interquartile range. Chi-squared tests were used to assess the differences in the number of products in different soft drink categories sold by individual companies over time. A Kruskal-Wallis test was used to test for differences between the sales-weighted mean sugar contents of each soft drink category—here, we weighted the analysis so that each brand had influence proportional to total sales, but the sum of the units of analysis was equal to the number of brands. We calculated the absolute mass of sugar sold and the sales-weighted mean sugar content of drinks sold over time.

## Results

In 2015, 55 soft drink companies sold 195 brands (Table 1). This rose to 57 companies and 199 brands in 2018. The total volume sales of soft drinks in the UK increased by 7% from 2015 to 2018, reaching 8.9 billion litres. Once adjusted for changes in the population size and expressed in per capita terms, the volume sales of soft drinks rose by 5%, from 351 ml per person per day to 367 ml per person per day.

**Table 1:** Data points in the sales volume and nutrient composition databases, 2015–2018

Description	2015	2016	2017	2018
Number of individual products in nutrient composition database	1966	1851	1725	1735
Number of brands in sales volume database	195	195	198	199
Number of companies in sales volume database	54	55	57	57
Total volume sales of soft drinks (million litres)	8336	8519	8728	8910
Volume sales of soft drinks (per capita per day)	351	356	362	367

**Fig. 1:**
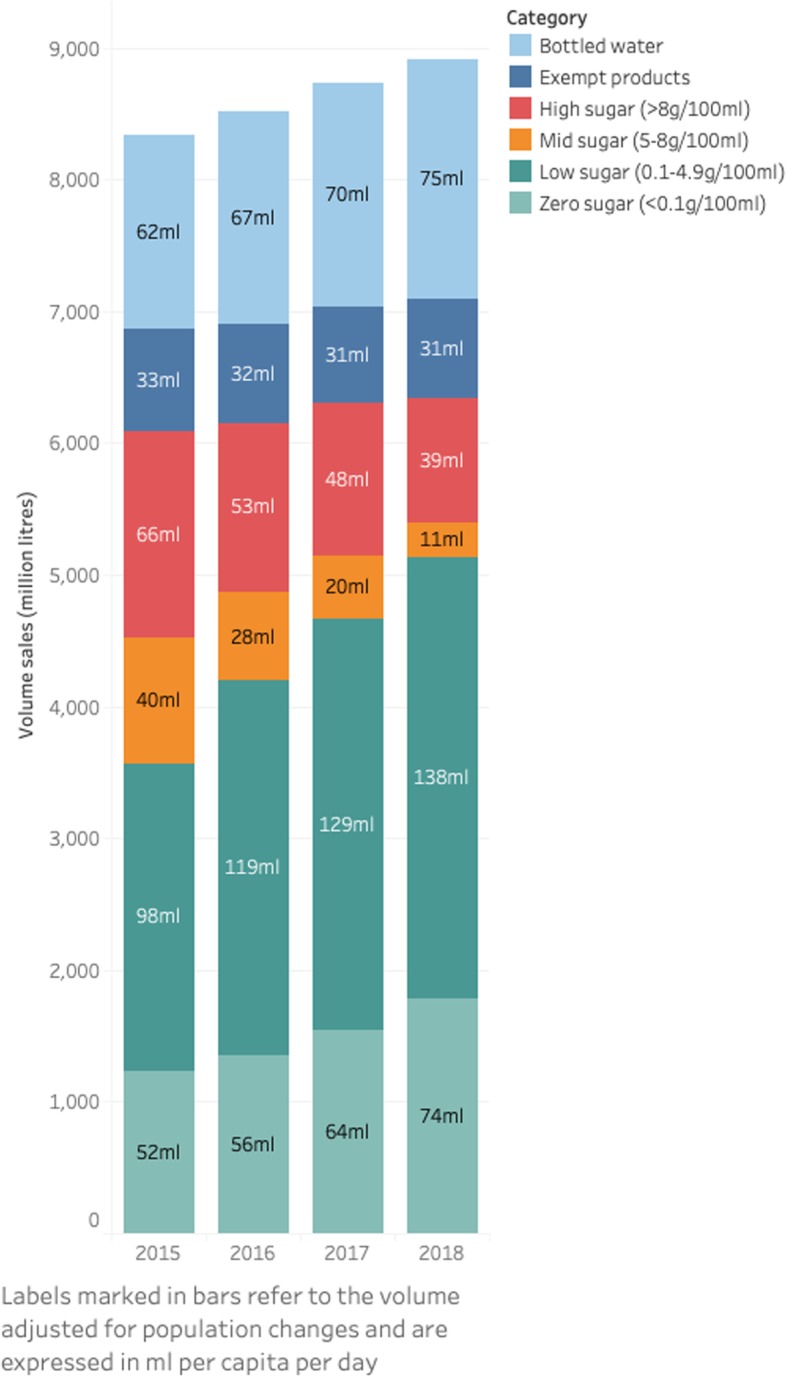
The volume sales of soft drinks by category, 2015–2018

There have been marked shifts in the proportion of drinks sold by sugar content. The total volume sales of high- and mid-sugar soft drinks included in the SDIL, with sugar content > 5 g/100 ml, fell from 31% in 2015 to 15% in 2018, equivalent to 106 ml per person per day to 50 ml per person per day. The combined low- and zero-sugar category increased from 43 to 48%, equivalent to 150 ml per person per day in 2015 to 212 ml in 2018 (Fig. 1). The total volume sales of bottled water and products exempt from the SDIL rose by 23%. This analysis was repeated without sales weighting (Additional file 1: Fig. S1).

The total volume of sugars sold from soft drinks decreased by 29%, from 368,000 t in 2015 to 261,000 t in 2015 (Table 2). The proportion of sugars sold from products included in the SDIL (i.e. high- and mid-sugar products) decreased from 63 to 44%, while the proportion increased for low-sugar products from 16 to 28%.

**Table 2:** The total volume of sugar sold from soft drinks, 2015–2018

Soft drink category	Total sugars (tonnes) sold by soft drink category			
	2015	2016	2017	2018
Total volume of sugars sold (t)	367,965 (100%)	328,685 (100%)	305,732 (100%)	261,324 (100%)
High-sugar (> 8 g/100 ml)	170,541.0 (46%)	139,192.3 (42%)	127,580.80 (42%)	102,553.10 (39%)
Mid-sugar (5–8 g/100 ml)	61,273.2 (17%)	43,325.3 (13%)	30,712.40 (10%)	13,948.90 (5%)
Low-sugar (0.1–5 g/100 ml)	58,739.3 (16%)	72,330.8 (22%)	76,015.40 (25%)	72,894.40 (28%)
Zero-sugar (< 0.1 g/100 ml)	0 (0%)	0 (0%)	0 (0%)	0 (0%)
Exempt products	77,411.6 (21%)	73,836.4 (22%)	71,423.10 (23%)	71,927.7 (28%)
Bottled water	0 (0%)	0 (0%)	0 (0%)	0 (0%)

Very similar trends were found, when expressed per capita per day, with the volume of sugars sold from soft drinks falling from 15.5 g per person per day in 2015 to 10.8 g in 2018, a reduction of 30% (Table 3).

**Table 3:** The volume of sugars sold by soft drink category, per capita per day, 2015–2018

Soft drink category	Volume of soft drink and sugars sold per capita per day			
	2015	2016	2017	2018
Volume of sugars sold from soft drinks (g/person/day)	15.5	13.7	12.7	10.8
High-sugar (> 8 g/100 ml)	7.2 (46%)	5.8 (42%)	5.3 (42%)	4.2 (39%)
Mid-sugar (5–8 g/100 ml)	2.6 (17%)	1.8 (13%)	1.3 (10%)	0.6 (5%)
Low-sugar (0.1–5 g/100 ml)	2.5 (16%)	3.0 (22%)	3.2 (25%)	3.0 (28%)
Zero-sugar (< 0.1 g/100 ml)	0.0	0.0	0.0	0.0
Exempt products	3.3 (21%)	3.1 (22%)	3.0 (23%)	3.0 (28%)
Bottled water	0.0	0.0	0.0	0.0

The absolute mean sugar content of soft drinks fell from 5.4 g/100 ml in 2015 to 3.9 g/100 ml in 2018, a reduction of 28% (Table 4). The annual changes from 2015 to 18 were − 7, − 10 and − 13%. The mean sugar content of the high- and mid-sugar drinks remained unchanged, but for the low-sugar category, the mean sugar content rose from 0.9 g/100 ml (0.5–3.2 g/100 ml) to 4.2 g/100 ml (0.9–4.7 g/100 ml).

**Table 4:** The average sugar content of soft drink products by category, 2015–2018

Soft drink category	Mean sugar content (g/100 ml) and IQR (Q2, Q1–Q3)				
	2015	2016	2017	2018	*p* value
Total soft drinks (g/100 ml)	5.4 (5.0, 0.2–10.0)	5.0 (4.3, 0.0–9.5)	4.5 (4.1, 0.0–8.9)	3.9 (3.8, 0.0–7.1)	< 0.01
High-sugar (> 8 g/100 ml)	10.8 (10.8, 10.0–11.8)	10.6 (10.6, 9.6–11.3)	10.6 (10.6, 9.9–11.0)	10.6 (10.6, 10.0–11.0)	0.18
Mid-sugar (5–8 g/100 ml)	6.7 (6, 6.0–7.3)	6.8 (6.8, 6.0–7.3)	6.7 (6.7, 5.9–7.2)	7.0 (7.0, 5.8–7.3)	0.94
Low-sugar (0.1–5 g/100 ml)	0.9 (0.9, 0.5–3.2)	1.1 (1.1, 0.5–3.8)	3.1 (3.1, 0.7–4.5)	4.2 (4.2, 0.9–4.7)	< 0.01
Zero-sugar (< 0.1 g/100 ml)	0.0 (0.0–0.0)	0.0 (0.0–0.0)	0.0 (0.0–0.0)	0.0 (0.0–0.0)	0.99
Exempt (g/100 ml)	10.0 (10.0, 8.7–11.0)	9.8 (9.8, 8.6–11.0)	9.8 (9.8, 8.6–11.0)	9.7 (9.7, 8.7–11.0)	0.21
Bottled water	0.0 (0.0–0.0)	0.0 (0.0–0.0)	0.0 (0.0–0.0)	0.0 (0.0–0.0)	0.99

Once adjusted for sales, the weighted mean sugar content of all soft drinks decreased significantly from 4.4 g/100 ml (IQR, 0.0–9.2 g/100 ml) to 2.9 g/100 ml (0.0–4.3 g/100 ml), an overall reduction of 34% (Table 5).

**Table 5:** Sales-weighted average sugar content of soft drinks by sugar content category, 2015–2018

Soft drink category	Mean sugar content (g per 100 ml) and IQR (Q2, Q1–Q3)				*p* value
	2015	2016	2017	2018	
Total soft drinks (g/100 ml)	4.4 (2.4, 0.0–9.2)	3.9 (1.8, 0.0–7.1)	3.5 (2.1, 0.0–6.1)	2.9 (1.2, 0.0–4.3)	< 0.01
High-sugar (> 8 g/100 ml)	11.0 (10.6, 10.6–11.0)	11.0 (10.6, 10.6–11.0)	11.0 (10.6, 10.6–11.0)	10.8 (10.6, 10.6–11.0)	0.58
Mid-sugar (5–8 g/100 ml)	6.4 (6.1, 5.7–7.3)	6.4 (6.3, 6.2–6.8)	6.5 (6.5, 5.9–7.4)	5.4 (5.2, 5.0–5.7)	0.15
Low-sugar (< 0.1–5 g/100 ml)	2.5 (2.3, 2.0–3.6)	2.4 (1.9, 1.4–3.9)	2.4 (2.1, 1.5–3.9)	2.2 (1.8, 1.1–2.9)	0.85
Zero-sugar (< 0.1 g/100 ml)	0.0 (0.0–0.0)	0.0 (0.0–0.0)	0.0 (0.0–0.0)	0.0 (0.0–0.0)	0.99
Exempt (g/100 ml)	10.0 (9.9, 9.6–10.2)	10.0 (9.7, 9.3–10.0)	9.7 (9.9, 9.2–10.4)	9.5 (10.0, 9.2–10.1)	0.75
Bottled water	0.0 (0.0–0.0)	0.0 (0.0–0.0)	0.0 (0.0–0.0)	0.0 (0.0–0.0)	0.99

**Fig. 2:**
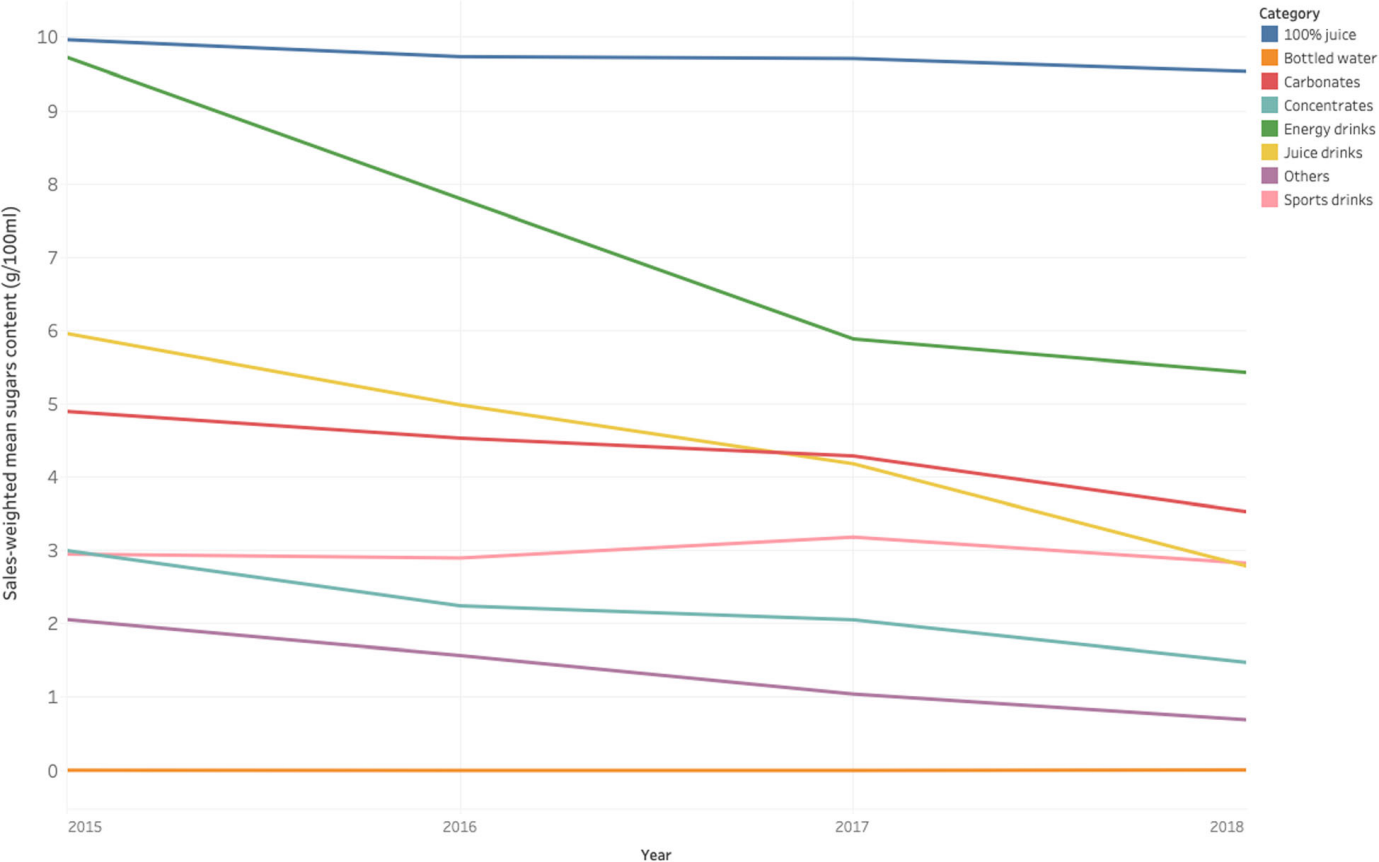
Sales-weighted mean sugar content of soft drinks by product type, 2015–2018

Energy drinks saw the greatest absolute reduction in sales-weighted mean sugar content (− 4.3 g/100 ml) (Fig. 2). Juice drinks also saw a significant reduction in sales-weighted mean sugar content (− 3.2 g/100 ml). The sales-weighted mean sugar content of carbonates, the category with the largest volume sales, fell by 29%. Changes in 100% juice and sports drinks were minimal.

**Fig. 3:**
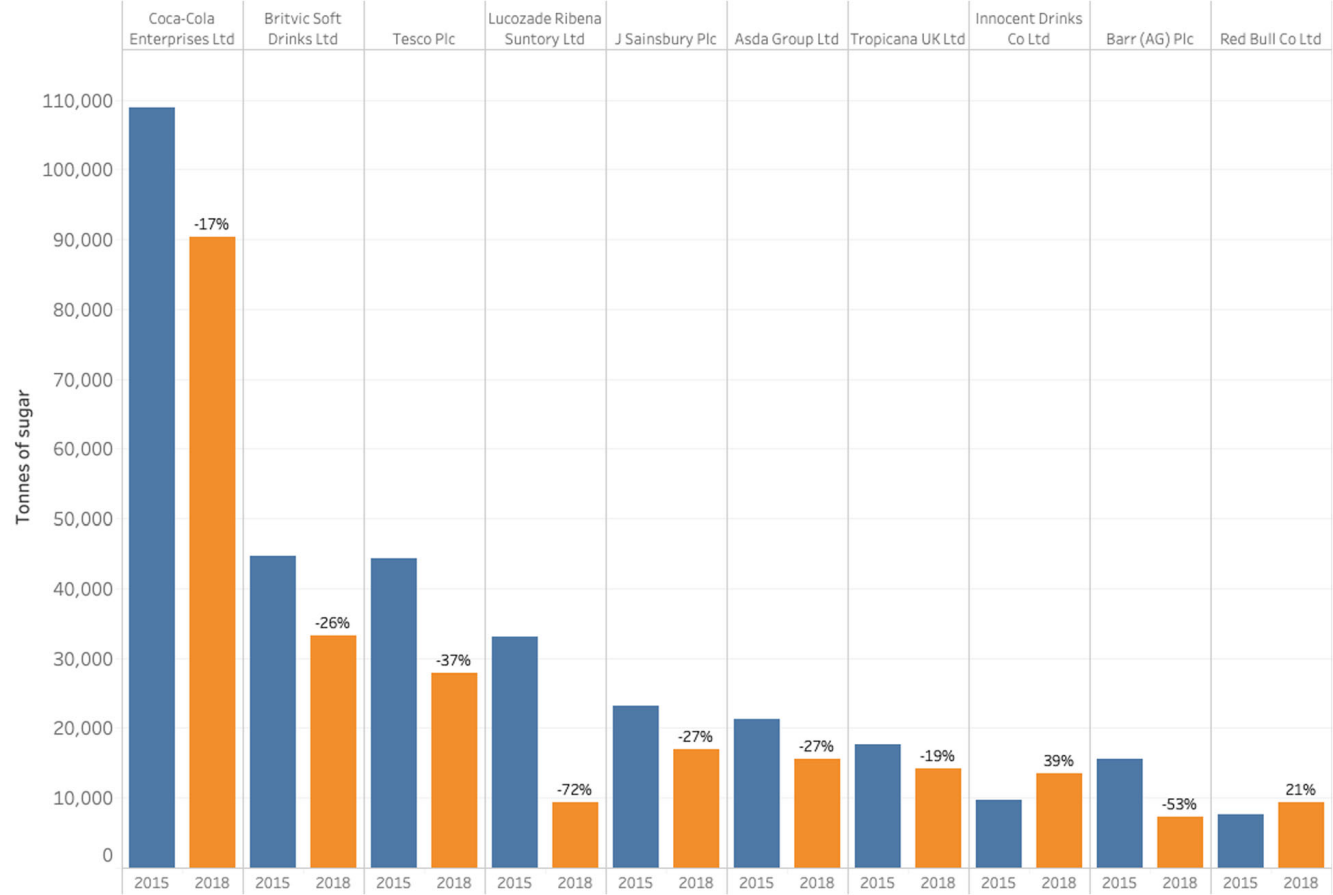
The total volume of sugars sold by top ten UK soft drink companies, 2015 and 2018

There was a decrease in the total volume sales of sugars for eight of the top ten companies (defined by their total contribution to total volume sales of sugars) (Fig. 3). The largest soft drinks company in terms of total volume sales of sugars in all four years was Coca-Cola; volume sales increased while the sugar content of products fell, leading to a 17% decrease in the total volume of sugars sold over time. Volume sales for Red Bull and Innocent increased with little change in the mean sugar content of their products, leading to increases in the volume sales of sugars for these two companies.

**Fig. 4:**
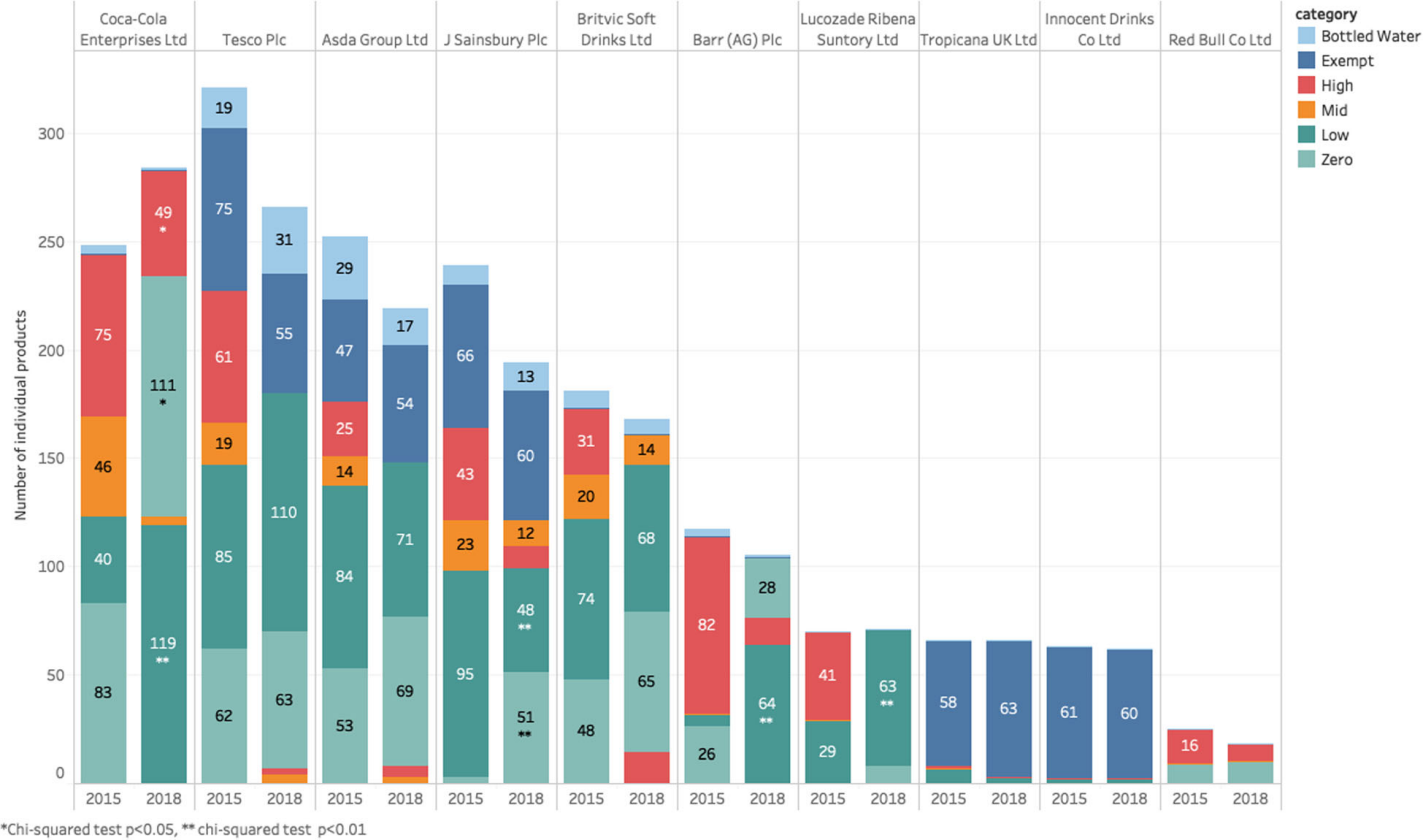
The availability of soft drinks by company, 2015 and 2018. Chi-squared test: *p < 0.05, **p < 0.01

There have been significant changes in the product portfolios of soft drink companies since 2015, including the reformulation of existing products and the introduction of new products. In 2015, eight of the top ten companies manufactured high- and mid-sugar products that are targeted by the SDIL, and two companies—Innocent and Tropicana—sold only exempt products (Fig. 4). Of these eight, six reformulated or removed more than 50% of the high- and mid-sugar products from their portfolios—leading to a 72% reduction in high- and mid-sugar products by 2018.

## Discussion

This analysis shows that despite a 5% increase in the volume sales of soft drinks in per capita terms, the sale of sugars from these products decreased by 30% between 2015 and 2018, equivalent to a reduction in 4.6 g sugar per capita per day. The mean sugar content of soft drinks declined by 34% throughout the period, with the greatest decrease in 2017–2018, the year the SDIL was implemented. There has been a 50% decrease in the volume sales of products that are subject to the SDIL. Six of the top ten companies have reformulated more than half the products in their portfolio during this period.

### Strengths and weaknesses of this study

We analysed the nutrient composition data of soft drink products across the whole market. By pairing composition data with sales data, we were able to capture not just what is available, but what is sold, and the net impact on total sugar sales. This provides novel insights into how both consumer demand for soft drinks and individual companies have responded to sugar reduction policies and the broader debate about sugar and soft drinks in the UK, and provides proof-of-concept for a method that could be used to monitor sugar reduction in other key food categories.

The nutrient composition data were collected from three supermarket websites (Asda, Sainsbury’s and Tesco) at single time points in the four years included in the analysis. The number of unique products included in this study is likely to be an underestimate of the total number of products on the market for two reasons. First, we have not included soft drinks that are only available for purchase outside of the three included supermarkets. Second, taking our data from single time points means that we have not captured the churn of soft drink products over the year (e.g. products leaving the marketplace and being replaced by new drinks, and seasonal fluctuations). Comparison of our dataset against other datasets used in the NIHR-funded SDIL Evaluation project [10] suggests that our datasets included the majority of drinks available in the UK marketplace. The nutrition information for brands that are not sold in the three supermarkets represented in the Brand View database (*n* = 12) had to be collected manually from the brand website in 2019. This means that no historical change could be measured for these brands, other than changes in sales levels. These products make a small contribution to overall sales (2% in 2018), and re-running the analysis to exclude these 12 brands made no substantive difference to the main results.

There are two main advantages to using sales data, as opposed to the national dietary survey data. Firstly, it avoids the reliance on individual recall of consumption, and secondly, the datasets include greater detail about individual brands. Euromonitor data has a wide coverage, including hypermarkets, supermarkets, convenience stores, vending machines and fast food outlets. These advantages, combined with the fact that it is an industry-accepted source of sales data, means that it is particularly suited to studying changes at a company and brand level. However, as with other possible data sources [11], there are limitations. The authors have no control over the data collection process and there is limited transparency in the methods of data collection or the reliability of the sources [11]. Euromonitor also classifies small and local brands under the umbrella term “others”. These brands represented 21% of total volume sales in 2018 but were excluded from this study. Within the Euromonitor dataset, the granularity by brand differed from company to company. For example, sales figures were given for Diet Coca-Cola and regular Coca-Cola separately, but for Sprite, both diet and regular versions were combined.

When multiple products were available under a single sales figure, we used an average sugar level from all identified products in the Brand View datasets. To test what impact this had on the results, we took the sales and composition data of those brands that did have a volume stratified between diet and regular versions (*n* = 16), combined the sales data and then calculated the mean sugar levels in the same way as for products where stratified sales data were not available. For these 16 brands, the mean sugar content was 30% lower overall when compared to the sales-weighted mean sugar content, with much heterogeneity by individual brand. This suggests that this limitation may bias the results presented here.

The lack of granularity in the sales data also meant that the changes in the sugar content of companies’ product portfolios provide only an overall picture, and we were not able to distinguish what proportion of the changes were due to the reformulation of existing products or the introduction of new low-sugar products to the market. The total UK population size was used to calculate the per capita figures. These results do not tell us anything about the distribution of soft drink sales within the population and will include a large percentage of zero consumers. This dataset, and other sources of food sales data, would benefit from the inclusion of population demographic factors in the future, including socioeconomic and geographic measures.

This analysis does not provide an assessment of the impact of the SDIL. Data were only available for four annual time points and were insufficient to analyse how sugar levels changed immediately before and after the announcement or introduction of the SDIL, or to estimate the specific impact of the SDIL in comparison with the general trend of sugar reduction in soft drinks. Inferences about the effect of the SDIL based on the increased rate of change coincident with the introduction of the SDIL should therefore be considered as tentative.

### Comparisons with other studies

This is the first study to report on the impact of calls to reduce sugar in the UK by individual companies. There is a global interest in benchmarking food company’s changes towards nutrition-related goals. Two such projects, the Access to Nutrition Index [12] and INFORMAS (International Network for Food, Obesity/NCD related Research, Monitoring and Action Support) Business Impact Assessment on Obesity tool [13], have used sales data from Euromonitor to estimate a “healthfulness score” for company’s portfolios using nutrient profiling. However, these scores cover the full range of a company’s products and actions and are not directly comparable with our quantitative analysis specifically of the soft drink market.

The findings we report here are similar to the few other studies that have looked at the sugar content of soft drinks in the UK in recent years, though only one other has reported on the change in sales.

A study of the sugar content in a sample of energy drinks in 2017 compared to 2015 (excluding zero-sugar products) reported a 10% decline (10.6 g/100 ml in 2015 vs 9.5 g/100 ml) [14]. Once adjusted to exclude zero-sugar products, our data shows the absolute mean content of energy drinks fell by 30% over the same time period, while the sales-weighted mean fell by 49% (9.6 g/100 ml in 2015 to 5.9 g/100 ml in 2017).

Public Health England (PHE) has also published data on changes in sugar from 2015 to 2018, based on the data provided by Kantar, a household panel survey [15]. We report a greater total volume of soft drink purchases (8.9 vs 3.9 billion litres in 2018) than PHE. The comparatively low volumes reported by PHE could be due to the underreporting of purchases by panel participants [16] or to overestimates of sales in Euromonitor data due to their indirect approach to estimation. Nonetheless, despite differences in the data sources, the findings are remarkably consistent. The average sales-weighted mean sugar content of soft drinks reported by PHE fell by 29% between 2015 and 2018 with a 10% increase in the total volume sales of soft drinks.

### Implications of this research

This study shows a 30% reduction in the sales of sugar from soft drinks in the last 4 years, equivalent to 4.6 g per capita per day. In order to better differentiate between the impact of changes in the sugar content of products and changes in purchasing behaviours, we repeated the analysis but kept the sugar content at 2015 levels. In this scenario, the total volume of sugars fell by only 4%. This suggests that the majority of the reduction in sugar sales was due to reformulation. Overall, volume sales have increased while the sugar content has declined, which highlights the opportunity for improvements in public health to be consistent with successful business practices—although we recognise that volume sales are only one indicator of a company’s success.

The rates of change in both mean sugar content and total sugar sales seem to have been accelerated by the announcement of the SDIL in March 2016 and its implementation in April 2018, and the sales of low-sugar products (< 5.0 g/100 ml) that are not subject to the levy have increased substantially. However, this paper is not an evaluation of the SDIL, and further work using a more granular time series is currently being conducted [10]. We show that six of the top ten soft drinks companies have reformulated more than 50% of SDIL-eligible products by 2018. Some companies reformulated all products, while others chose to maintain the sugar content of key brands. There was no change in the sugar content of brands excluded from the SDIL. This evidence suggests that the implementation of the levy acted as an extra incentive for companies to reformulate above and beyond what was already being done as part of the voluntary or consumer-driven reformulation.

The marked increase in the proportion of drinks in the market classified as low sugar is encouraging. However, the volume of sugar sold from products classified as “low-sugar” has increased by 24% from 2015 to 2018, and the sugar content of many of these products is close to the threshold for the levy. This suggests that reducing the amount of sugar that makes it permissible to classify as a low-sugar drink (and therefore untaxed) may prompt further reductions. The proportion of sugar sold from tax-exempt products, e.g. 100% juices, has increased (although the total volume has declined by 7%). Consideration could be given to including these products in the SDIL to encourage reductions in the sugar content and to signal clearly to consumers that these are high-sugar products.

Applying this method of combining sales data with the nutrient composition of products to other key food categories included in the sugar and calorie reduction targets would provide policymakers with a comprehensive assessment of the rate of change in the sales of sugar. Providing greater transparency about individual company’s responses would also enable government, non-government organisations and investors to benchmark companies, and highlight those who are making the most, or least, change towards public health nutrition goals.

## Conclusion

This analysis provides strong evidence of reductions in the sugar content of soft drinks, together with the changes in product portfolios and consumer purchasing patterns which have led to a substantial reduction in the sales of sugar in soft drinks.

## Supplementary information

**Additional file 1: Figure S1.** Number of individual soft drink products by category, 2015-2018.

## Data Availability

The data that support the findings of this study are available from Euromonitor International and Brand View, but restrictions apply to the availability of these data, which were used under licence for the current study, and so are not publicly available. Data however are available from the authors upon reasonable request and with permission from Euromonitor International and Brand View.
