# Supplementary figures and images for: Reductions in sugar sales from soft drinks in the UK from 2015 to 2018
Source: BMC Med. 2020 Jan 13;18:20. doi: 10.1186/s12916-019-1477-4 (PMC6956503; doi:10.1186/s12916-019-1477-4)

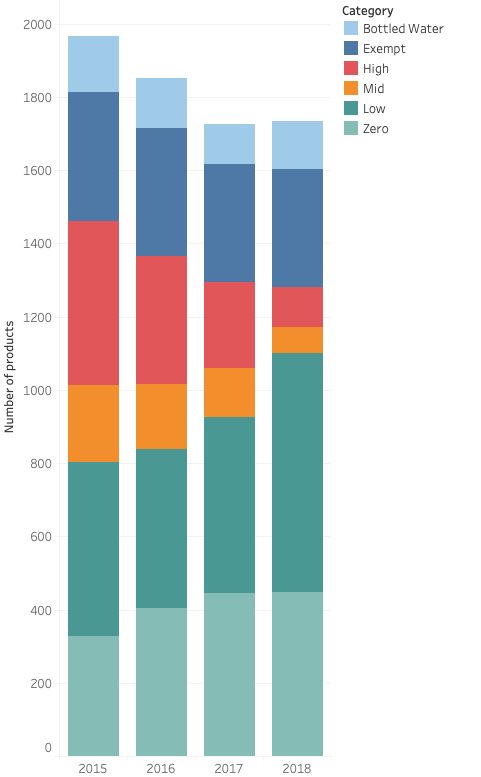

Supplement: Supplementary file 1 — Additional file 1: Figure S1. Number of individual soft drink products by category, 2015-2018. [file 12916_2019_1477_MOESM1_ESM.png]
